# Time from pre‐labor rupture of membrane at term to delivery in grand multiparous women

**DOI:** 10.1002/ijgo.70953

**Published:** 2026-03-13

**Authors:** Shai Ram, Michael Lavie, Roy Bitan, David Nadav Sabag, Dotan Madar, Anat Lavie, Yariv Yogev, Roza Berkovitz Shperling

**Affiliations:** ^1^ Lis Hospital for Women's Health, Tel Aviv Sourasky Medical Center, Affiliated to the Gray Faculty of Medicine and Health Science Tel Aviv University Tel Aviv Israel

**Keywords:** grand multiparity, labor progression, parity, pre‐labor rupture of membranes (PROM), vaginal delivery

## Abstract

**Objective:**

The effect of parity on labor progression following term pre‐labor rupture of membranes (PROM) has been described, but detailed patterns among grand multiparous women remain unclear. Traditional labor models show accelerated labor with increasing parity, yet few studies have specifically examined PROM latency distributions or heterogeneity within high‐parity groups. The aim of the present study was to characterize labor progression after term PROM across parity groups, with specific focus on identifying heterogeneity and distinct distributional patterns among grand multiparous women.

**Methods:**

This was a retrospective cohort study including 11 219 women with singleton, cephalic pregnancies at ≥37 weeks gestation who underwent expectant management following term PROM. Women were stratified into three groups: nulliparous (*n* = 4347), multiparous parity 1–4 (*n* = 6725), and grand multiparous parity ≥5 (*n* = 147). The primary outcome was PROM‐to‐delivery interval analyzed by mean, median, and percentile distribution. Secondary outcomes included mode of delivery and second‐stage duration.

**Results:**

Mean PROM‐to‐delivery interval decreased with increasing parity (nulliparous 13.4 ± 7.1 h vs multiparous 10.4 ± 6.3 h vs grand multiparous 10.2 ± 6.5 h; *P* < 0.001). While grand multiparous women had labor kinetics comparable to multiparous women at lower percentiles, a biphasic pattern emerged: beyond the 70th percentile, latency in grand multiparous women plateaued and resembled nulliparous progression. Grand multiparous women had the shortest second stage (14.3 ± 9.8 min) and highest spontaneous vaginal delivery rate (96.6%).

**Conclusion:**

Beyond confirming known parity‐related differences in labor duration after term PROM, this study demonstrates clinically relevant heterogeneity within the grand multiparous population. A distinct subgroup shows delayed progression that is not captured by mean or median estimates. Distributional, percentile‐based assessment may refine counseling and support more individualized timing of intervention following term PROM.

## 1 INTRODUCTION

Pre‐labor rupture of membranes (PROM) at term occurs in approximately 8% of term pregnancies.^1^, ^2^, ^3^, ^4^ For women at term who present with PROM, labor induction is generally recommended if spontaneous labor does not commence shortly after rupture, with an observation period of 12 to 24 h often considered reasonable during expectant management.^5^, ^6^, ^7^, ^8^

The progression of spontaneous labor varies considerably with parity, as multiparous typically experience more rapid cervical dilation and shorter labor durations than their nulliparous counterparts.^12^ A recent cohort study of approximately 2000 women with PROM reported that nulliparous women were less likely to progress to spontaneous labor within the first 24 h compared to multiparous women.^9^, ^10^, ^11^

While the association between parity and labor duration following term PROM is well established,^11^, ^12^ prior studies have largely relied on comparisons of central tendency and have treated grand multiparous women as a homogeneous group. Data on within‐group heterogeneity and distributional patterns of labor progression in this population are scarce. In the present study, we aimed to characterize the full distribution of PROM‐to‐delivery intervals among grand multiparous women using a percentile‐based approach and to explore whether distinct subpatterns of labor kinetics exist within this group.

## 2 MATERIALS AND METHODS

### 2.1 Study design and setting

This was a retrospective, single‐center cohort study conducted at a single university‐affiliated tertiary medical center with approximately 12 500 deliveries annually between December 2011 and January 2023. The study was approved by the local Institutional Review Board (IRB) committee (TLV‐0284‐08). As this was a retrospective study using existing data, the Institutional Review Board approved a waiver of informed consent. The ethical approval number covers a broader departmental protocol retrospective data.

### 2.2 Study population

Women with singleton term pregnancies (≥37^+0^ weeks' gestation) who presented with spontaneous PROM and were not in active labor were eligible for inclusion. PROM was diagnosed by clinical history and confirmed by sterile speculum examination, and baseline assessment of dilation, effacement, and fetal station. Our institutional protocol defined active labor as ≥4 cm with regular contractions, consistent with local practice at the time.

Local institutional protocol recommends labor induction and/or augmentation in cases of term PROM. Nonetheless, women who expressed a preference for expectant management were allowed to proceed conservatively for up to 24 h, contingent upon meeting the clinical criteria of clear amniotic fluid (absence of meconium staining or bloody amniotic fluid), absence of clinical chorioamnionitis (defined as maternal fever ≥38°C, uterine tenderness, or a leukocyte count ≥18 000/μL), and reassuring fetal heart rate monitoring. During the expectant management period, repeat pelvic examinations were avoided unless clinically indicated (e.g., progression of contractions or non‐reassuring fetal heart rate).

Women diagnosed with PROM who were not suitable for expectant management underwent immediate induction or augmentation were excluded. The women who comprised the study cohort were stratified by parity into three groups: nulliparous, multiparous (1–4 previous births), and grand multiparous (≥5 previous births).

### 2.3 Clinical management

Pelvic examinations were avoided during expectant management unless contractions became more painful or frequent. They were carried out in a sterile manner as labor progressed or if non‐reassuring fetal heart rate patterns had been identified. Fetal heart rate monitoring was performed, and maternal vital signs were monitored every 4 h. Complete blood count, including white blood cell count, were assessed at admission and repeated every 24 h or as clinically indicated. Antibiotic prophylaxis was administered immediately upon admission according to GBS status or at 18 h from ROM if GBS status was unknown or if women developed an intrapartum fever ≥38°C.

### 2.4 Study procedures and data collection

The data were retrieved from electronic medical records. Maternal characteristics included maternal age, body mass index (BMI, calculated as weight in kilograms divided by height in meters squared), gestational weight gain, smoking status, and the need for artificial reproductive treatments. Labor assessment included cervical examination at admission (effacement and dilatation) and committed fetal station. The labor assessment represented the first cervical examination at admission. Time intervals were recorded from PROM to delivery, with separate documentation of the second stage and third stage (delivery to placental expulsion). Re‐amniotomy was defined as an additional artificial rupture of membranes performed after the initial diagnosis of PROM in order to facilitate labor progression.

### 2.5 Outcome measures

The primary outcome was the time interval from documented PROM to spontaneous delivery, including mean, median, and percentile times, stratified by parity groups (nulliparous, multiparous, and grand multiparous). Secondary outcomes included mode of delivery (normal vaginal delivery or vacuum extraction), duration of second and third stages of labor, and rates of re‐amniotomy.

Active labor was defined as cervical dilation ≥4 cm with regular contractions. Committed fetal station was defined as a fetal head station ranging from −3 to +3 on vaginal examination, indicating that the presenting part is engaged and descending into the maternal pelvis. Pre‐labor fetal head engagement was defined as a fetal head station of ≥−2 on vaginal examination at the first cervical assessment upon admission.

These definitions were not used to determine the primary outcome (PROM‐to‐delivery interval) and therefore does not materially affect the main analyses.

### 2.6 Statistical analysis

Statistical analyses were performed using SPSS version 29 (IBM Corp., Armonk, New York). Data distribution was assessed using the Shapiro–Wilk test and visual inspection of histograms. Continuous variables were compared between the three parity groups by means of one‐way analysis of variance (ANOVA) with Tukey's post‐hoc test for normally distributed variables or Kruskal–Wallis test with Dunn's post hoc test for non‐normally distributed variables. Categorical variables were compared using the chi‐square test or Fisher exact test. Post hoc pairwise comparisons were performed with Bonferroni correction for multiple comparisons.

Time‐to‐delivery data were analyzed using both parametric and non‐parametric methods. Percentiles of the PROM‐to‐delivery interval were calculated using the empirical cumulative distribution function, with 95% confidence intervals (CIs) generated by bootstrap resampling. This percentile‐based approach allowed us to determine the time by which a specific proportion of women remained undelivered after PROM, similar in concept to survival analysis. For example, the 50th percentile represents the time by which 50% of the women had delivered, while the 70th or 90th percentiles reflect the longer durations required for the remaining undelivered cases. This analysis aimed to capture differences in labor progression dynamics across the entire distribution, beyond the central tendency reflected by mean or median values. In addition, Kaplan–Meier survival curves were used to visualize the cumulative proportion of undelivered women over time, stratified by parity group. The results are presented as means ± standard deviation (SD), medians (interquartile range [IQR], or numbers (percentages)). A *P* value less than 0.05 was considered statistically significant, with Bonferroni correction applied for multiple comparisons.

## 3 RESULTS

**FIGURE 1:**
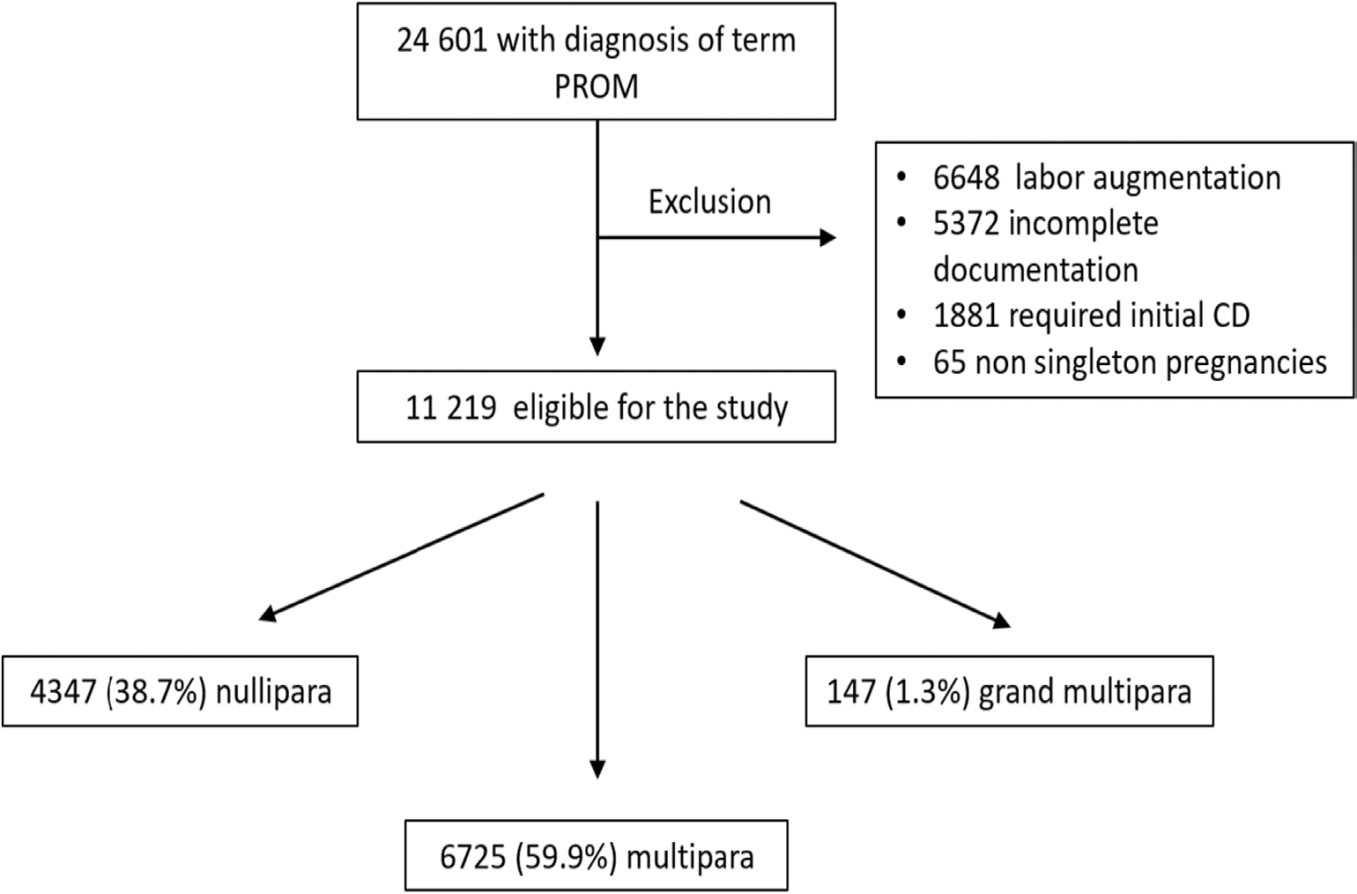
Study population flow diagram.

Out of 154 427 deliveries, 24 601 (15.9%) women were diagnosed with PROM during the study period. Of these, 11 219 (45.6%) met the inclusion criteria, comprising 4347 (38.7%) nulliparous, 6725 (59.9%) multiparous, and 147 (1.3%) grand multiparous women (Figure 1).

There were significant demographic and clinical differences across the study groups, such as maternal age and BMI, which increased significantly with parity (age: 30.85 ± 4.39 vs 34.16 ± 4.41 vs 36.79 ± 4.21 years; BMI: 22.03 ± 4.24 vs 22.62 ± 4.39 vs 25.23 ± 5.43, both *P* < 0.001) (Table 1).

**TABLE 1:** Baseline maternal and obstetric characteristics by parity group.

Characteristic	Nulliparous (*n* = 4347)	Multiparous (*n* = 6725)	Grand multiparous (*n* = 147)	*P* value
Maternal age (years), mean ± SD	28.6 ± 4.9	31.4 ± 5.1	34.7 ± 4.2	<0.001
BMI, mean ± SD	24.8 ± 3.9	26.1 ± 4.2	27.4 ± 4.6	<0.001
Gestational age at PROM (weeks), mean ± SD	39.2 ± 1.1	39.3 ± 1.0	39.4 ± 1.0	0.12
Neonatal birth weight (g), mean ± SD	3145.6 ± 371.0	3301.5 ± 384.6	3428.6 ± 382.8	<0.001
Diabetes (GDM/PGDM), *n* (%)	142 (3.3%)	254 (3.8%)	7 (4.8%)	0.41
Hypertension, *n* (%)	68 (1.5%)	92 (1.4%)	3 (2.0%)	0.73
Epidural analgesia, *n* (%)	3475 (79.9%)	3718 (55.3%)	46 (31.3%)	<0.001
Re‐amniotomy, *n* (%)	683 (15.7%)	742 (11.0%)	13 (8.8%)	<0.001

*Note*: *P* values calculated using ANOVA for continuous variables and Chi‐square test for categorical variables, as appropriate. Data are presented as mean ± SD or *n* (%), unless otherwise indicated. BMI, calculated as weight in kilograms divided by height in meters squared.

Abbreviations: BMI, body mass index; GDM, gestational diabetes mellitus; PGDM, pregestational diabetes mellitus; PROM, pre‐labor rupture of membranes; SD, standard deviation.

At admission, nulliparous women were more likely to present with pre‐labor fetal head engagement, defined as a fetal head station of ≥−2 on vaginal examination at the first cervical assessment upon admission with PROM, compared with multiparous and grand multiparous women (94.7% compared to 89.3% and 83.3% of the multiparous and the grand multiparous women, respectively, *P* < 0.001). The rate of known GBS colonization was significantly higher among the grand multiparous women (26.2% compared with 13.1% and 12.7% for the nulliparous and multiparous women, respectively, *P* < 0.001).

Neonatal birth weight increased with parity (3145.6 ± 371.0 g in nulliparous, 3301.5 ± 384.6 g in multiparous, and 3428.6 ± 382.8 g in grand multiparous women; *P* < 0.001; Table 1), although the absolute differences between groups were modest and within normal birth weight range across all groups.

Delivery outcomes demonstrated distinct parity‐related patterns as shown in Table 1. The grand multiparous women exhibited shorter durations of the second stage of labor compared to the nulliparous and multiparous women (14.29 ± 23.96 min vs 66.05 ± 50.34 and 22.43 ± 30.51, respectively, *P* < 0.001) and the lowest incidence of need to preform re‐amniotomy (0.7% vs 3.6% and 3.1%, *P* = 0.037). Grand multiparous and multiparous women showed higher rates of spontaneous vaginal delivery (96.6% and 96.8% vs 88.6%, *P* < 0.001). The number of assisted vaginal delivery rates decreased by parity (nulliparous 9.6% vs multiparous 2.6% vs grand multiparous 2.0%, *P* < 0.001).

A detailed analysis of PROM‐to‐delivery time intervals revealed similar mean durations between grand multiparous and multiparous women, with longer intervals observed among nulliparous women (13.39 ± 11.26 vs 10.22 ± 10.00 and 10.42 ± 11.25 h, respectively; *P* < 0.001; Table 2).

**TABLE 2:** Labor and delivery outcomes by parity group.

Outcome	Nulliparous	Multiparous	Grand multiparous	*P* value
PROM‐to‐delivery time (h), mean ± SD	13.4 ± 7.1	10.4 ± 6.3	10.2 ± 6.5	<0.001
Second stage (min), mean ± SD	66.1 ± 44.2	22.4 ± 18.7	14.3 ± 9.8	<0.001
Spontaneous vaginal delivery, *n* (%)	3852 (88.6%)	6518 (96.8%)	142 (96.6%)	<0.001
Operative vaginal delivery, *n* (%)	312 (7.2%)	87 (1.3%)	1 (0.7%)	<0.001
Cesarean delivery, *n* (%)	183 (4.2%)	120 (1.8%)	4 (2.7%)	<0.001

*Note*: *P* values calculated using ANOVA for continuous variables and Chi‐square test for categorical variables, as appropriate. Data are presented as mean ± SD or *n* (%), unless otherwise indicated.

Abbreviations: PROM, pre‐labor rupture of membranes; SD, standard deviation.

**FIGURE 2:**
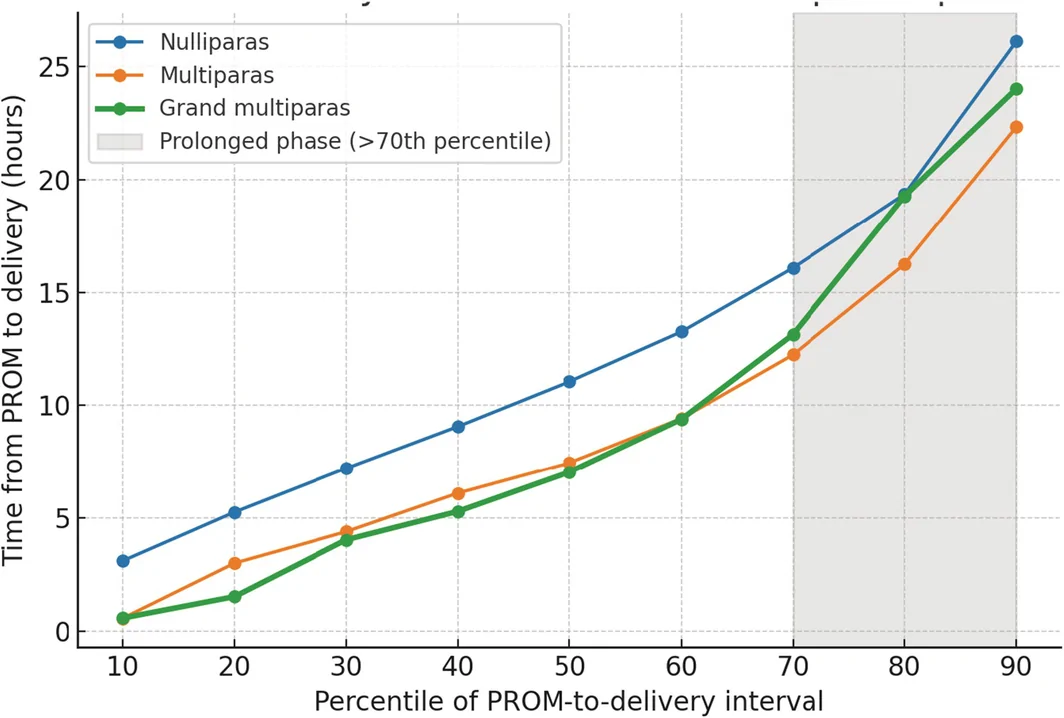
Time from premature rupture of membrances (PROM) to delivery analyzed by percentiles and stratified by parity groups.

To further evaluate labor dynamics, a percentile‐based analysis of the cumulative distribution of time from PROM to delivery was conducted. Among grand multiparous women, a biphasic pattern emerged: up to the 60th percentile of cumulative deliveries (i.e., the point at which 60% of grand multiparas had delivered), the PROM‐to‐delivery interval resembled that of multiparous women. However, beyond the 70th percentile, labor progression slowed, and the time to delivery began to approximate that observed in nulliparous women (Table 3; Figure 2).

**TABLE 3:** Percentile distribution of PROM‐to‐delivery interval (h).

Percentile	Nulliparous	Multiparous	Grand multiparous
10th	4.2	3.6	3.7
25th	7.1	5.9	6.0
50th (median)	12.6	9.7	9.6
70th	15.8	13.8	14.0
90th	22.3	19.4	21.7

Abbreviation: PROM, pre‐labor rupture of membranes.

## 4 DISCUSSION

The present study provides new insight into labor physiology following term PROM, demonstrating a previously unrecognized biphasic progression pattern among grand multiparous women.

The principal findings of this large retrospective study are as follows. First, mean and median PROM‐to‐delivery intervals were longest among nulliparous women, shorter among multiparous women, and shortest—though nearly identical—between multiparous and grand multiparous women. Second, despite this overall similarity, a percentile‐based analysis revealed a distinct biphasic labor pattern in grand multiparous women: while the majority progressed similarly to multiparous women, those undelivered after the 70th percentile demonstrated prolonged latency resembling nulliparous labor. Third, labor outcomes—including shorter second‐stage duration and higher spontaneous vaginal delivery rates—were most favorable among grand multiparous women, although their PROM latency distribution was unexpectedly heterogeneous.

These findings refine the established understanding that increasing parity is consistently associated with faster labor progression. Traditional labor models, beginning with Friedman's curve and followed by contemporary analyses by Zhang et al., demonstrated clear parity‐related differences in cervical dilation rates and total labor duration.^9^, ^10^ However, neither model stratified women by very high parity thresholds nor evaluated those with PROM specifically. Prior PROM‐focused studies showed that nulliparous women are less likely to enter spontaneous labor within 24 h compared with multiparous women, but they grouped higher‐parity women together and did not interrogate distributional heterogeneity or biphasic patterns.^8^, ^11^, ^13^ To our knowledge, no previous investigation has characterized time‑to‑delivery distributions in grand multiparous women after PROM using a percentile‑based approach.

Several physiological mechanisms may underlie the biphasic labor pattern observed in grand multiparous women. First, once active labor is established, greater pelvic tissue compliance and cervical responsiveness may contribute to the shorter second stage and higher spontaneous vaginal delivery rates, aligning with prior data in high‑parity cohorts.^14^ Conversely, the prolonged PROM‑to‑delivery intervals beyond the 70th percentile suggest that a subset experiences delayed onset or progression of active labor. This delayed pattern may be influenced by age‑related myometrial changes, higher body mass index, or altered oxytocin receptor dynamics, factors associated with slower labor kinetics in observational studies.^11^, ^15^ In addition, lower rates of pre‑labor head engagement documented in this cohort among grand multiparous women could contribute to mechanical inefficiency—for example with asynclitism or occiput posterior position—phenomena known to prolong labor even among women with previous births.^10^, ^11^ Finally, cumulative cervical remodeling after multiple prior births may result in variable readiness for labor and heterogeneity in latency duration.

Moreover, the grand multiparous women in our cohort were older, had a higher BMI, and exhibited increased rates of anemia. These characteristics have been independently associated with altered labor kinetics,^4^, ^8^ ultimately resulting in a labor pattern that resembles that of nulliparous women. Although neonatal birth weight differed statistically across parity groups, the absolute differences were small (approximately 150–280 g) and all group means fell within normal birth weight range, making less likely that birth weight meaningfully confounded the observed differences in labor progression patterns.

The clinical implications are two‐fold. First, parity‑based expectations alone may be insufficient to guide counseling and timing after term PROM. While most grand multiparous women proceed to delivery within expected timeframes, those undelivered beyond the 70th percentile may represent a subgroup at risk for protracted latency. In such cases, earlier clinical reassessment—for cervical status, fetal head position, and evolving signs of dystocia—and timely augmentation could be considered rather than a uniform policy of expectant management. Second, the favorable delivery outcomes we observed among grand multiparous women support the safety of expectant management in well‑selected patients, but clinicians should maintain vigilance when percentile thresholds are exceeded, as delayed progression may represent emerging labor dysfunction rather than reassuring kinetics.

Strengths of this study include its large sample size, long study period, and homogeneous inclusion of only women undergoing expectant management after term PROM, which together allow characterization of natural labor progression across parity groups. The percentile‑based analytic framework is another strength, revealing clinically relevant heterogeneity that would be obscured by means or medians alone. Standardized institutional PROM protocols during the study period further reduce practice variation and support valid comparisons among groups. Limitations include the retrospective design and potential selection bias (women choosing expectant management may differ from those undergoing induction), the relatively small number of grand multiparous women limiting precision at higher percentiles.

In addition, key intrapartum variables that may influence labor kinetics—such as fetal head position, cervical consistency, uterine contraction patterns, and real‐time oxytocin dosing—were not systematically captured and could not be accounted for in the analyses. Although the institutional definition of active labor during the study period differed from contemporary guideline thresholds, this did not affect the primary outcome of PROM‐to‐delivery interval. Finally, unmeasured confounding inherent to observational studies cannot be fully excluded. Prospective multicenter studies with standardized labor assessments are needed to validate these findings and to clarify the mechanisms underlying prolonged latency in a subset of grand multiparous women.

Future research should identify predictors of prolonged latency among grand multiparous women after PROM to enable early recognition and individualized care. Prospective studies incorporating standardized assessment of cervical status, fetal position (including sonographic occiput orientation), and uterine activity are warranted. Evaluation of tailored augmentation strategies triggered by percentile thresholds could determine whether earlier intervention improves efficiency without increasing maternal or neonatal morbidity.

## 5 CONCLUSION

Grand multiparous women with term PROM demonstrate a previously unrecognized biphasic labor progression pattern. Although most progress similarly to multiparous women, a distinct subset shows delayed progression resembling nulliparous labor beyond the 70th percentile. These findings refine current understanding of parity‑related labor physiology and support percentile‑based counseling and decision making after PROM. Prospective validation and mechanism‑focused research are needed to inform individualized management strategies.

## AUTHOR CONTRIBUTIONS

Shai Ram: Design, planning, conduct, data analysis, and manuscript writing. Michael Lavie: Design, planning and manuscript writing. Roy Bitan: Design, planning and manuscript writing. David Nadav Sabag: Design, planning and manuscript writing. Dotan Madar: Planning and manuscript writing. Anat Lavie: Planning and manuscript writing. Yariv Yogev: Design, planning and manuscript writing. Roza Berkovitz Shperling: Design, planning, data analysis and manuscript writing.

## CONFLICT OF INTEREST STATEMENT

The authors do not have any conflicts of interest.

## Data Availability

Research data are not shared.
